# Tuning the Acid–Base Properties of Lignin-Derived Carbon Modulated ZnZr/SiO_2_ Catalysts for Selective and Efficient Production of Butadiene from Ethanol

**DOI:** 10.3390/molecules28186632

**Published:** 2023-09-15

**Authors:** Na Liu, Yingluo He, Kangzhou Wang, Fei Chen, Jie Yao, Guohui Yang, Shufang Huang, Lishu Shao, Noritatsu Tsubaki

**Affiliations:** 1 Ministry of Forestry Bioethanol Research Center, School of Materials Science and Engineering, Central South University of Forestry and Technology, Changsha 410004, China; liuna1912@163.com; 2 Department of Applied Chemistry, School of Engineering, University of Toyama, Gofuku 3190, Toyama 930-8555, Japan; yingluo@eng.u-toyama.ac.jp (Y.H.); kangzhou_wang@163.com (K.W.); d2078103@ems.u-toyama.ac.jp (F.C.); yaojie@eng.u-toyama.ac.jp (J.Y.); thomas@eng.u-toyama.ac.jp (G.Y.); 3 Department of Environmental Monitoring, College of Changsha Environmental Protection, Changsha 410004, China; huangshufan@163.com

**Keywords:** lignin, ethanol, butadiene, ZnZr/SiO_2_ catalysts, acid–base properties

## Abstract

The direct selective conversion of ethanol to butadiene (ETB) is a competitive and environmentally friendly process compared to the traditional crude cracking route. The acid–base properties of catalysts are crucial for the direct ETB process. Herein, we report a rationally designed multifunctional lignin-derived carbon-modulated ZnZr/SiO_2_ (L-ZnZr/SiO_2_) catalyst with suitable acid–base properties for the direct ETB reaction. A variety of characterization techniques are employed to investigate the relationship between the acid–base properties and catalytic performance of the multifunctional lignin-modulated ZnZr/SiO_2_ catalysts. The results revealed that the rationally additional lignin-modulated carbon enhances both the acidity and basicity of the ZnZr/SiO_2_ catalysts, providing a suitable acid–base ratio that boosts the direct ETB reactivity. Meanwhile, the 1% L-ZnZr/SiO_2_ catalyst possessed ethanol conversion and butadiene selectivity as high as 98.4% and 55.5%, respectively, and exhibited excellent catalytic stability.

## 1. Introduction

The production of fuels and chemicals from green, renewable biomass is receiving more and more attention to alleviate environmental issues and fossil depletion [1,2]. Among them, ethanol, as an important C_2_ platform molecule, mainly comes from biomass fermentation. It can be converted into high-value-added chemicals such as butanol, ethylene oxide, ethyl acetate, butadiene (BD), aromatics, and liquid fuels [3,4]. BD, as an important industrial chemical, has been widely used as a monomer in the manufacture of synthetic resins, nylon, and rubber [5]. Currently, more than 90% of BD is produced as a co-product with ethylene and propylene from the naphtha thermal steam cracking process [6]. However, the depletion of petroleum resources and the emergence of alternative raw materials for ethylene production, such as shale gas, seriously threaten the supply of BD [7]. Therefore, the development of the direct conversion of ethanol to butadiene is a competitive and promising route from a green, economical, and sustainable perspective [8,9].

As a very complex multi-step tandem reaction, the direct ETB reaction requires the coordination of multiple active centers, including Lewis acid sites and base sites [10]. Therefore, rationally designed catalysts with suitable acid–base properties would play a decisive role in achieving a selective and efficient production of butadiene. In addition, the reaction conditions of each stage have an effect on the yield of BD. Therefore, it is necessary to improve the properties and structure of the catalyst and optimize the process conditions to improve its activity and stability [11,12,13]. At present, different Zn precursors are often used to modulate the acid–base properties of Zr/SiO_2_-based catalysts in the process of direct conversion of ethanol to butadiene because Zn species are not only the active centers for dehydrogenation of ethanol but also interact with silanol groups to form strong Lewis acid sites [14]. However, Zn addition cannot effectively counterpoise each active center and affects the dehydrogenation activity of ethanol. Studies have shown that the acid–base properties can also be effectively adjusted by adding mixed metal oxides (MgO, CuO, Ta_2_O_5_, Y_2_O_3_, La_2_O_3_, etc.) [15,16,17].

Notably, Jing and co-workers have reported an ultrathin hydrothermal carbon (HTC)-shelled Mo_2_C@C material with abundant active sites, fast transport pathways, and stable reaction interfaces due to the abundant functional groups of biomass-derived carbon [18,19,20]. Lignin, as an abundant and renewable aromatic natural polymer with oxygen-rich functional groups, can be used as a raw material to synthesize hydrothermal carbon with multiple functional groups [21,22,23]. Meanwhile, biomass-derived hydrothermal carbon can also effectively modulate the acid–base properties of catalysts by functional group structure [24,25]. To our knowledge, using lignin-derived carbon to modulate the acid–base properties of ZnZr/SiO_2_-based catalysts for maximized production of butadiene from ethanol has not been reported.

In this work, we report an efficient multifunctional lignin-derived carbon-modulated ZnZr/SiO_2_ catalyst with suitable acid–base properties that exhibited outstanding catalytic performance in the direct ETB reaction. The characterization results revealed that lignin-derived carbon provides suitable acid–base properties for better synergies with multi-active component centers. Moreover, the relationship between acid–base properties and catalytic performance was discussed.

## 2. Results

### 2.1. Structural Characterization

**Figure 1:**
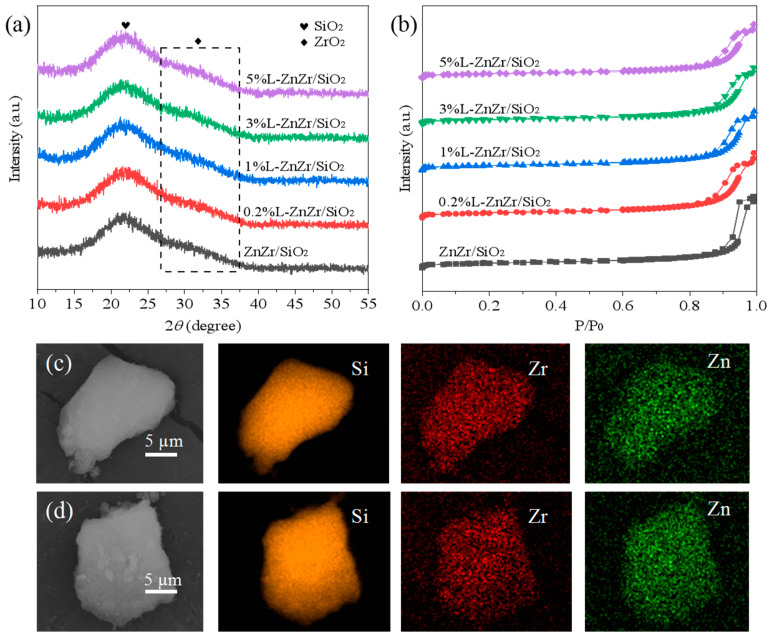
Structural characterization of the catalysts. XRD patterns of the catalysts (**a**); N_2_ adsorption–desorption isotherms (**b**); FE-SEM images and EDS elemental mapping images of ZnZr/SiO_2_ (**c**) and 1%L-ZnZr/SiO_2_ (**d**).

**Figure 2:**
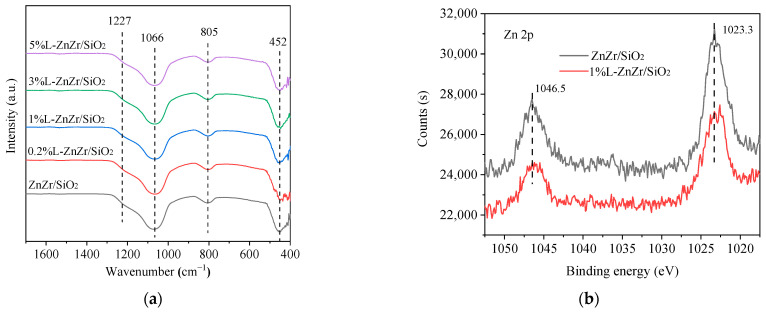

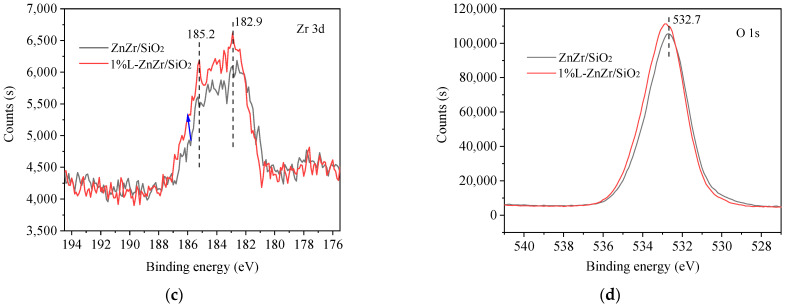
Functional properties of the catalysts. (**a**) FT-IR spectra of the synthesized catalysts; (**b**) Zn 2p region for ZnZr/SiO_2_ and 1%L-ZnZr/SiO_2_ catalysts; (**c**) Zr 3d region for ZnZr/SiO_2_ and 1%L-ZnZr/SiO_2_ catalysts; (**d**) O 1s region for ZnZr/SiO_2_ and 1%L-ZnZr/SiO_2_ catalysts.

The XRD patterns of all catalysts are presented in Figure 1a. The broad diffraction peak at 15–40° belonged to amorphous silica [26]. The catalysts exhibit weak shoulder peaks in the range of 27 to 38°. Combined with the analysis of the FT-IR in Figure 2a, there is a band near 1076 cm^−1^, which is due to the interaction between the Zr species and silanols, forming Si-O-Zr [27,28]. However, no characteristic peak of ZnO or ZrO_2_ was detected on all samples, suggesting that the Zn and Zr species were well-dispersed on SiO_2_ support and that their crystal sizes were smaller than the XRD detection limit [29].

The pore distributions of five catalysts were evaluated by N_2_ adsorption–desorption isotherms (Figure 1b). According to the International Union of Pure and Applied Chemistry (IUPAC) [30], all the catalysts belonged to class IV (a) adsorption isotherms, indicating that the catalysts had a typical mesoporous structure. However, the isothermal hysteresia type ZnZr/SiO_2_ tends to be H1 and H2, while m%L-ZnZr/SiO_2_ is similar to H2 and H5. H5 types are associated with pore structures that have both open and partially blocked mesopores. This change may be related to the blockage of pores caused by the adhesion of carbon. The pore properties are summarized in Table S1. The average diameter of the mesopores (Dmeso) of the m%L-ZnZr/SiO_2_ catalysts was slightly reduced. The specific area (S_BET_), total volume (V_total_), and mesoporous volume (V_meso_) decreased with the increase in the amount of lignin, while the average diameter of the micropores (D_micro_) and the micropore volume (V_meso_) did not change obviously.

The morphology and elemental distribution of the catalysts were analyzed by FE-SEM and EDS elemental mapping analysis, and the results are shown in Figure 1c,d and Figure S1. With the increase in lignin, the surface of the catalysts gradually became rougher. The EDS elemental mapping images of the ZnZr/SiO_2_ and 1%L-ZnZr/SiO_2_ catalysts showed that the Zn and Zr species were uniformly dispersed on the silica supports (Figure 1c,d). In addition, as shown in Figure S2, after heat treatment, the color of the catalysts gradually changed from white to dark gray with the increase in lignin content.

### 2.2. Functional Group Properties

FT-IR spectra were used to analyze the functional groups of the catalysts. As shown in Figure 2a, the broad bond at 1066 cm^−1^ belonged to the typical asymmetric stretching vibrations of Si-O-Si bonds in the silica matrix [31]. Two characteristic peaks at 805 and 452 cm^−1^ were attributed to the symmetric stretching and rocking vibrations of Si-O-Si bonds, respectively [27]. The FT-IR spectra results indicated that the lignin treatment did not change the typical Si-O-Si bonds or the functional groups of the catalysts.

The surface chemical states of the catalysts were investigated using XPS analysis. The Si 2p peak at 103.4 eV as the internal standard to calibrate binding energy was more accurate [28,32]. The Zn 2p peaks of the ZnZr/SiO_2_ and 1%L-ZnZr/SiO_2_ catalysts appeared at 1046.5 and 1023.3 eV, respectively, and the Zr 3d peaks appeared at 185.2 and 182.9 eV (Figure 2b,c), suggesting that the bonds of Si-O-Zn and Si-O-Zr were formed in ZnZr/SiO_2_ and 1%L-ZnZr/SiO_2_, respectively [33,34]. The O 1s peak of 1%L-ZnZr/SiO_2_ displayed higher binding energies than those of ZnZr/SiO_2_ (Figure 2d). The Zr 3d peak of 1%L-ZnZr/SiO_2_ shifted to higher binding energies compared to the ZnZr/SiO_2_ catalyst (Figure 2c). These results are due to the adsorption and surface interaction between lignin-derived carbon and ZnO, ZrO_2_, and SiO_2_. By comparing the yields of butadiene production from ethanol using the ZnZr/SiO_2_ and 1%L-ZnZr/SiO_2_ catalysts, which are 38.3% and 43.9%, respectively, it can be inferred that the introduction of an appropriate amount of lignin-derived carbon into the ZnZr/SiO_2_ catalyst enhances the production of butadiene from ethanol [33].

### 2.3. Acid–Base Properties

**Figure 3:**
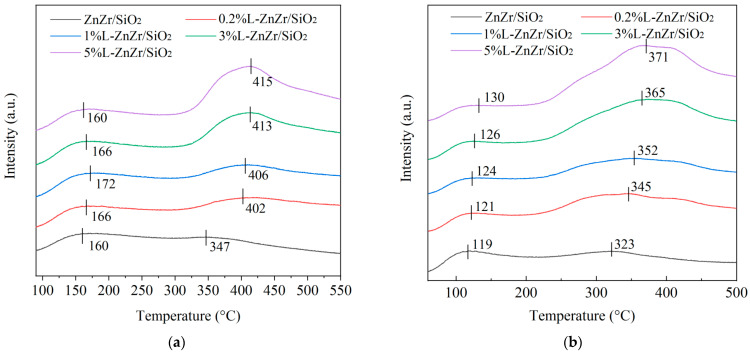

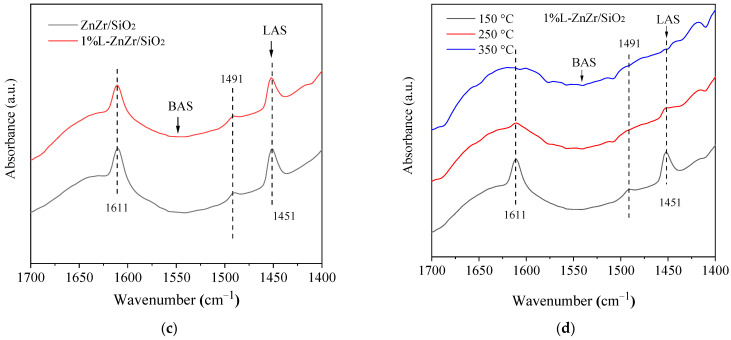
Acid–base properties of the catalysts. (**a**) NH_3_-TPD profiles of the synthesized catalysts; (**b**) CO_2_-TPD profiles of the synthesized catalysts; (**c**) FT-IR spectra of adsorbed pyridine on ZnZr/SiO_2_ and 1%L-ZnZr/SiO_2_ catalysts after desorption at 150 °C; (**d**) FT-IR spectra of adsorbed pyridine on 1%L-ZnZr/SiO_2_ catalysts after desorption at 150, 250, and 350 °C.

A NH_3_-TPD analysis was used for measuring the acid properties of the catalysts, and the results are shown in Table 1 and Figure 3a. ZnZr/SiO_2_ exhibited two peaks at 160 °C and 347 °C, which were attributed to the adsorption of NH_3_ molecules from weak and strong acid (Sa) sites, respectively. Compared with the ZnZr/SiO_2_ catalyst, the strong acid peaks of the m%L-ZnZr/SiO_2_ catalysts shifted to a higher temperature, indicating that the strong acid strength of the catalyst increased after lignin-derived carbon addition [35,36]. Meanwhile, with the increase in lignin, the weak acid (Wa) amount first increased and then decreased, while the strong acid amount increased gradually.

Py-IR was used to further investigate the types of acid sites. In Figure 3c, the peaks at 1611 and 1451 cm^−1^ were assigned to the interaction between pyridine and Lewis acid sites, and the peak at 1491 cm^−1^ was attributed to the Lewis and Brønsted acid sites [37]. No Brønsted acid sites were detected on the ZnZr/SiO_2_ and 1%L-ZnZr/SiO_2_ catalysts; only Lewis acid sites were observed. The number of Lewis acid sites in the two catalysts is listed in Table 1. ZnZr/SiO_2_ possessed a higher number of Lewis acid sites than 1%L-ZnZr/SiO_2_. In Figure 3d, it can be observed that when the pyridine FT-IR desorption temperature is higher than 250 °C, the peak intensity declines dramatically. These results indicate that 1%L-ZnZr/SiO_2_ has a smaller number of Lewis acid sites, which probably promotes the aldol condensation and MPV reduction steps in the direct ETB process [38].

CO_2_-TPD was employed to analyze the basic properties of the catalysts, and the results are summarized in Table 2 and Figure 3b. All five samples exhibited two kinds of CO_2_ desorption peaks. The low-temperature peaks were assigned to weak basic sites (Wb’s), and the high-temperature peaks were attributed to strong basic sites (Sb’s). With the increase in lignin, the weak and strong basic peaks shifted to high temperatures, suggesting an enrichment in the strength of the basic sites. The number of basic sites is listed in Table 2. Compared with ZnZr/SiO_2_, the weak basic amount of m%L-ZnZr/SiO_2_ catalysts declined, while the strong base amount increased. With the increase in lignin, the weak basic amount first decreased and then increased. These events proved that the additional lignin-derived carbon modulated the basic properties of ZnZr/SiO_2_ effectively.

### 2.4. Catalytic Performance

The catalytic performances of the ZnZr/SiO_2_ and m%L-ZnZr/SiO_2_ catalysts for the direct ETB conversion were investigated, and the results are compared in Table 3. The ZnZr/SiO_2_ catalyst exhibited 82.5% ethanol conversion and about 46% butadiene selectivity. The ethanol conversion increased to a maximum value of 84.1%, and a high BD selectivity of 52.2% was achieved in the 1%L-ZnZr/SiO_2_ sample at 375 °C. Meanwhile, the butadiene yield and STY reached 43.9% and 198 g·kg_cat_^−1^·h^−1^, respectively. However, when lignin further increased to 5% in the catalyst, the ethanol conversion and butadiene selectivity dropped sharply, and other byproducts increased. These results demonstrate that an appropriate amount of lignin can greatly improve the reaction performance of the ZnZr/SiO_2_ catalyst, while excessive lignin might cover part of the active sites and inhibit the occurrence of cross-aldol condensation, one of the side reactions.

**Figure 4:**
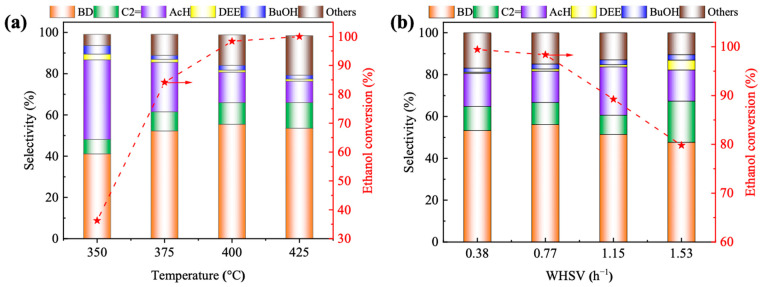
Effect of the reaction temperature (**a**) and WHSV (**b**) on catalytic performance of the direct ETB reaction on the 1%L-ZnZr/SiO_2_ catalyst. Reaction conditions: catalyst = 0.25 g, T = 400 °C, WHSV = 0.77 h^−1^, and TOS = 6 h. Ethanol, butadiene, ethylene, acetaldehyde, diethyl ether, and 1-butanol are abbreviated as EtOH, BD, C2=, AcH, DEE, and BuOH, respectively.

As far as we know, the direct ETB process is a complex tandem reaction that needs multiple activation energies. The reaction conditions, such as temperature and ethanol–catalyst contact time, have important influences on its catalytic performance. To further optimize the catalytic performance of the 1%L-ZnZr/SiO_2_ catalyst, various reaction temperatures and the WHSV were evaluated, and the results are shown in Figure 4 and Table S2. With the increase in the reaction temperature from 350 to 425 °C, the ethanol conversion and the selectivity of BD, ethylene, and other byproducts increased continuously, while the selectivity of acetaldehyde and diethyl ether declined obviously, which certified that the cross-aldol condensation of acetaldehyde with croton aldehyde occurred at high temperatures [39]. At the same time, the butadiene selectivity and butadiene yield increased to a maximum value of 55.5% and 54.6%, respectively, at 400 °C (Table S2). Therefore, considering butadiene selectivity, the reaction temperature of 400 °C was chosen as the optimal temperature to further investigate the effect of the WHSV on catalytic performance with the 1%L-ZnZr/SiO_2_ catalyst. When we enhanced the WHSV from 0.38 to 1.53 h^−1^, the 1%L-ZnZr/SiO_2_ catalyst achieved the highest STY (as high as 296 g·kg_cat_^−1^·h^−1^) at the WHSV of 1.15 h^−1^ (Table S3). As shown in Figure 4b, with the increase in WHSV, the conversion of ethanol and other byproducts diminished while the selectivity of acetaldehyde and diethyl ether increased, suggesting that the aldol condensation required a lower WHSV in this tandem reaction [40].

### 2.5. Relationship between Acid–Base Properties and Catalytic Performance

**Figure 5:**
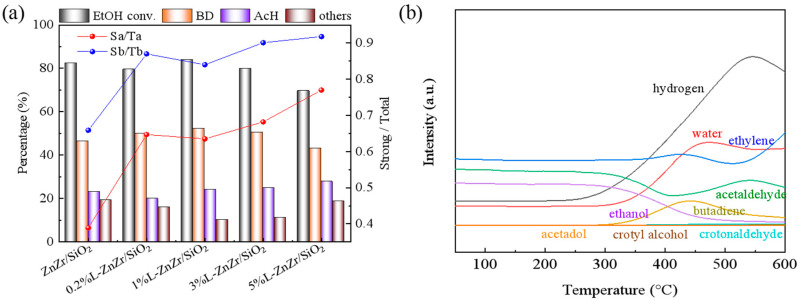
(**a**) Relationship between acid–base properties and catalytic performance for direct ETB conversion; (**b**) ethanol-TPSR profiles for direct ETB conversion in the 1%L-ZnZr/SiO_2_ catalyst.

From the characterization of the catalysts and the reaction performance results, we can infer that the acid–base properties of catalysts have an important influence on the catalytic performance of the direct ETB conversion [26,41]. A relationship between the acid–base properties of the catalyst and the catalytic performance was established to maximize the production of butadiene from ethanol. As shown in Figure 5a, as the lignin content increased from 0.2% to 5%, the butadiene selectivity and butadiene yield increased to a maximum value with 1% lignin addition and then decreased with a further increase in lignin up to 5%. The acetaldehyde selectivity decreased with the increase in lignin. The 3%L-ZnZr/SiO_2_ and 5%L-ZnZr/SiO_2_ catalysts exhibited higher byproduct selectivities, indicating that the side reactions such as the cross-aldol condensation of acetaldehyde with crotonaldehyde were intensified when compared to those of the aldol condensation of acetaldehyde. The 1%L-ZnZr/SiO_2_ catalyst possessed the highest ethanol conversion (84.1%) and butadiene selectivity (52.2%) of all five catalysts at 375 °C and a WHSV of 0.77 h^−1^, and it had a moderate ratio of acid to total acid and basic to total basic. Compared to other carbon-modulated catalysts, the best catalyst (1%L-ZnZr/SiO_2_) had the smallest strong-acid-to-total-acid ratio (Sa/Ta = 0.64) and the largest weak-acid-to-total-acid ratio (Wa/Ta = 0.37), whereas the ratio of strong base to total base was the smallest (Sb/Tb = 0.84), and the ratio of weak base to total base was the largest (Wb/Tb = 0.16). This is because strong acid sites can easily lead to the side reactions of ethanol dehydration to produce ethylene and ether, while weak acids are beneficial for the dehydration reactions in the third and fifth steps of the main reaction. However, being too alkaline can lead to excessive condensation during the second step of aldol condensation to form highly polymerized products during C–C coupling. These analyses are consistent with the corresponding reaction results. Thence, a suitable acid–base property of the catalyst was essential to enhance the aldol condensation of acetaldehyde and maximize the production of butadiene from ethanol.

The main reaction pathway of the direct ETB conversion was investigated by ethanol-TPSR. In the 1%L-ZnZr/SiO_2_ catalyst, when the reaction temperature increased, ethanol started decreasing at 250 °C while hydrogen and ethylene increased at the same time, suggesting that ethanol dehydrogenation occurred (Figure 5b). Subsequently, BD appeared leisurely at temperatures over 300 °C, and acetaldehyde showed an opposite trend, indicating that the acetaldehyde generated by the ethanol dehydrogenation reaction participated quickly in the subsequent aldol condensation reaction. Then, the BD signal started decreasing when the temperature reached over 425 °C, indicating that more byproducts were formed by generating acetaldehyde and crotyl alcohol through cross-aldol condensation reactions. Unfortunately, only acetaldehyde was detected in ethanol-TPSR, while other important intermediates, such as acetaldol, crotonaldehyde, or crotyl alcohol, were not detected. These phenomena suggested that other trace intermediates such as acetaldol, crotonaldehyde, or crotyl alcohol may have participated in subsequent reactions immediately after their formation. These results demonstrate that the aldol condensation of acetaldehyde was a rate-determining step and that the synergy of multiple active sites effectively boosted butadiene synthesis from ethanol.

### 2.6. Stability and Spent Catalyst Characterization

**Figure 6:**
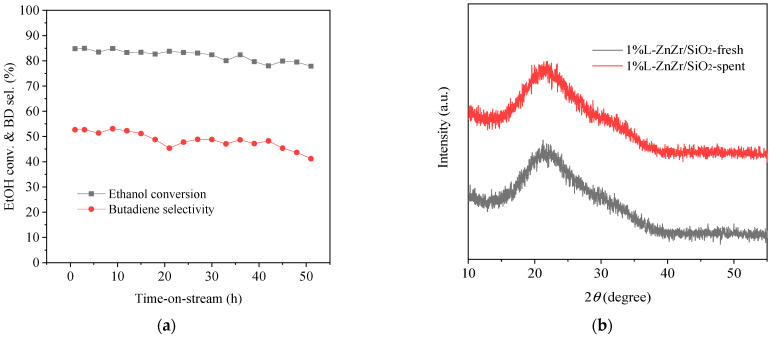

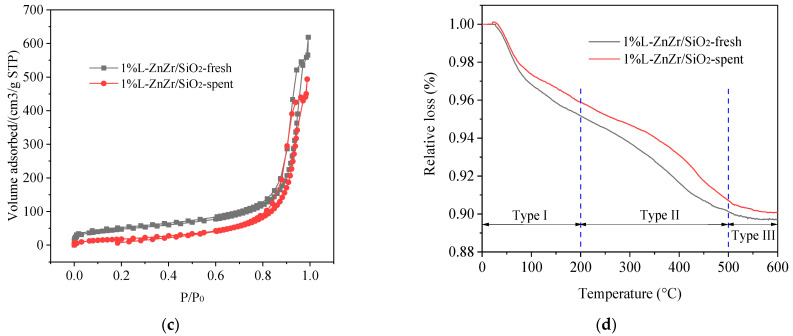
Stability and characterization of fresh and spent catalysts. (**a**) Stability of 1%L-ZnZr/SiO_2_ for the direct ETB conversion. Reaction conditions: catalyst = 0.25 g, T = 375 °C, WHSV = 0.77 h^−1^, and TOS = 50 h. Ethanol and butadiene are abbreviated as EtOH and BD; (**b**) XRD pattern of the fresh and spent catalyst of 1%L-ZnZr/SiO_2_; (**c**) N_2_ adsorption–desorption isotherms of the fresh and spent catalyst of 1%L-ZnZr/SiO_2_; (**d**) TG analysis of the fresh and spent catalyst of 1%L-ZnZr/SiO_2_.

A long-term stability experiment was performed on the 1%L-ZnZr/SiO_2_ catalyst, and the results are shown in Figure 6a. The ethanol conversion and butadiene selectivity remained relatively stable for over 50 h in the reaction test. The ethanol conversion and the selectivity of BD declined slightly from 84.8 to 77.9% and from 52.7 to 41.2%, respectively. To further study the deactivation mechanism of the catalyst, we characterized the spent catalysts with various characterization techniques. In Figure 6b, compared with the fresh 1%L-ZnZr/SiO_2_ catalyst, the XRD pattern of the spent 1%L-ZnZr/SiO_2_ catalyst shows no obvious difference. However, the N_2_ adsorption–desorption isotherms of the fresh and spent catalysts are demonstrably changed (Figure 6c). The specific surface area and total volume of the spent 1%L-ZnZr/SiO_2_ catalyst decreased (Table S4), which may be due to some mesopores of the 1%L-ZnZr/SiO_2_ catalyst being partially blocked by the high carbon products and carbon deposits. A further TG analysis showed that the first stage (type I: 0–200 °C) primarily involves volatile substances such as water. In the second stage (type II: 200–500 °C), the primary process involves the thermal decomposition or carbonization of carbonaceous compounds within the sample. It is obvious that the spent 1%L-ZnZr/SiO_2_ catalyst showed a significant weight loss at around 450 °C compared with the fresh 1%L-ZnZr/SiO_2_ catalyst, indicating that the high carbon products and carbon deposits were formed during the long-term stability test of the direct ETB reaction. In the third stage (type III: above 500 °C), there is minimal thermal change, suggesting that the carbonaceous deposits have largely decomposed or completed carbonization.

## 3. Materials and Methods

### 3.1. Catalysts Preparation

A ZnZr/SiO_2_ catalyst with a molar ratio of 0.02 Zn: 0.05 Zr: 1.0 Si was prepared using the wet-impregnation method. In brief, 0.20 g of zinc nitrate (Zn(NO_3_)_2_·6H_2_O, 99.0 wt%, Wako Pure Chemical Corporation, Osaka, Japan) and 0.45 g of zirconyl nitrate dihydrate (ZrO(NO_3_)_2_·2H_2_O, 97.0 wt%, Wako Pure Chemical Corporation) were dissolved in 30 mL of water to form a homogeneous solution. Then, 2.0 g of fumed SiO_2_ powder (0.2–0.3 μm, Sigma-Aldrich Co., LLC., St. Louis, MO, USA) was added to this solution and stirred for 6 h. The water was then evaporated to dryness at 120 °C, and the obtained solid powder was calcined at 500 °C in the air for 5 h with a temperature ramp of 5 °C/min. The calcined sample was denoted as ZnZr/SiO_2_.

Lignin-derived carbon-modulated ZnZr/SiO_2_ catalysts were fabricated using the facile heat-treatment method. Typically, the calculated amount of lignin (71~80%, Tokyo Chemical Industry Co., Ltd., Tokyo, Japan) was added to the ZnZr/SiO_2_ catalyst and ground for 30 min. Subsequently, the sample was transferred into a Teflon-lined stainless-steel autoclave and treated at 120 °C for 24 h, followed by calcination at 450 °C in a N_2_ atmosphere to obtain lignin-derived carbon-modulated ZnZr/SiO_2_ catalysts. The obtained samples were named m%L-ZnZr/SiO_2_, where m (0.2, 1, 3, 5) stands for the preset weight percentage of lignin in the whole catalyst.

### 3.2. Catalyst Characterization

X-ray powder diffraction (XRD) patterns were measured by using a RINT 2400 X-ray diffractometer (Rigaku, Tokyo, Japan) with Ni-filtered CuKα radiation at a scanning speed of 2 °·min^−1^ in the range of 10–80°.

Specific surface area, pore volume, and pore size were determined by nitrogen (N_2_) adsorption–desorption measurements on a 3Flex 2MP instrument (Micromeritics, Norcross, GA, USA). All the samples were pretreated at 200 °C under vacuum conditions for 6 h. The total surface area was calculated based on the BET equation, the micropore distribution was evaluated using the t-plot method, and the mesopore distribution was evaluated from the adsorption isotherm using the Barrett–Joyner–Halenda (BJH) method.

The crystal morphology and elemental mapping were analyzed by a field-emission scanning electron microscope (FE-SEM, JEOL, Tokyo, Japan) using a JSM-6700F equipped with energy dispersive spectroscopy (EDS).

X-ray photoelectron spectroscopy (XPS) was used to analyze the surface properties of catalysts on the ESCALAB 250Xi instrument (Thermo Fisher Scientific, Waltham, MA, USA). All binding energies were calibrated with the Si 2p line at 103.4 eV.

Fourier transform infrared resonance (FT-IR) was obtained on an IR Prestige-21 (GC, Tokyo, Japan)infrared spectrometer ranging from 400 to 4000 cm^−1^.

The acid and basic properties of the catalysts were analyzed by temperature-programmed desorption of ammonia (NH_3_-TPD) and carbon dioxide (CO_2_-TPD) on a BELCATII-T-SP instrument (Microtrac mrb, York, PA, USA), respectively. In brief, a catalyst of 50 mg was pretreated at 400 °C for 1 h under a helium atmosphere (50 mL·min^−1^) to remove water and other impurities. Then, the sample was subjected to NH_3_ adsorption (5 vol % NH_3_/He) or pure CO_2_ adsorption under a flow of 30 mL·min^−1^ at 50 °C for 1 h, followed by degassing of physisorbed NH_3_ or CO_2_ under helium at 50 °C for 1 h. Finally, the NH_3_ or CO_2_ desorption profiles were recorded from 50 to 500 °C at a heating rate of 10 °C·min^−1^.

The acid type of the catalyst was identified by pyridine-adsorption infrared spectroscopy (Py-IR) using a Bruker Tensor 27 instrument (Bruker, Mannheim, Germany). Typically, the sample was pretreated under vacuum conditions at 400 °C for 1 h to remove water adsorbed on the catalyst. The samples were then adsorbed to pyridine vapors at 100 °C for 10 min, followed by degassing of the physisorbed pyridine molecules under a vacuum for 30 min. Then, the spectra were recorded at 150 °C, 250 °C, and 350 °C.

Temperature-programmed surface reaction (TPSR) was performed on a BELCATII-T-SP instrument (MicrotracBEL, Osaka, Japan) connected with a mass spectrometer (MS, MICROTRAC MRB). In brief, a sample of 0.05 g was placed in the reactor and pretreated under helium flow (20 mL·min^−1^) at 450 °C for 1 h, and then the sample was cooled to 30 °C. Then, the saturated vapor of ethanol was continuously introduced into the reactor using helium as the carrier gas, and the catalyst was heated from 30 to 500 °C at a ramp rate of 5 °C·min^−1^. The trends of various important intermediates were monitored by MS.

The operation process for ethanol-TPD was similar to that of ethanol-TPSR. After the catalyst was pretreated, saturated ethanol vapor was adsorbed instead of continuously feeding ethanol. The important intermediates, such as ethanol (C_2_H_5_OH, 46), hydrogen (H_2_, 2), water (H_2_O, 18), acetaldehyde (C_2_H_5_OH, 46), butadiene (C_4_H_6_, 54), acetaldol (C_4_H_8_O_2_, 88), crotonaldehyde (C_4_H_6_O, 70), crotyl alcohol (C_4_H_8_O, 72), ethylene (C_2_H_4_, 28), and ether (C_4_H_10_O, 74), were monitored using MS.

Thermogravimetric (TG) analysis of the coke deposition of spent catalyst using a Shimadzu DTG-60 thermal analyzer (GC, Tokyo, Japan) was carried out. The sample was heated from room temperature to 600 °C with a heating rate of 10 °C·min^−1^ under an air atmosphere.

### 3.3. Catalytic Activity Measurement

**Scheme 1:**
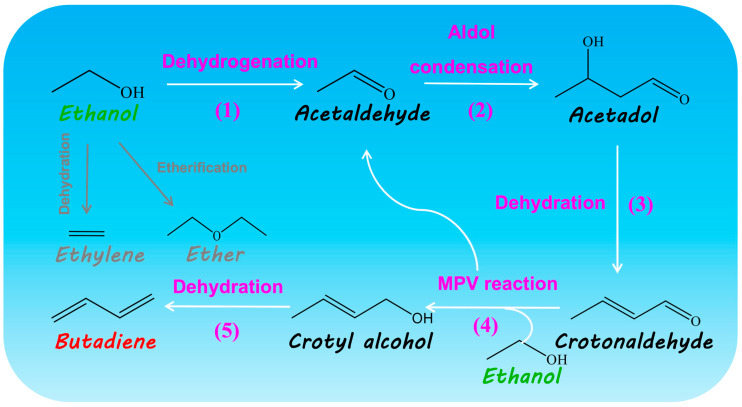
Main reaction pathway of the process of direct conversion of ethanol to butadiene and (1)–(5) represents the sequence of steps of the reaction.

The main reaction pathway of this multi-step tandem reaction involved five key steps, as shown in Scheme 1: (1) acetaldehyde is formed via ethanol dehydrogenation on basic or redox sites [42]; (2) acetaldehyde is transformed into acetaldol by aldol condensation at Lewis acid sites; (3) acetadol is dehydrated to crotonaldehyde on silanol groups [43,44]; (4) transformation of crotonaldehyde with ethanol into crotyl alcohol via the Meerwein–Ponndorf–Verley (MPV) reaction at Lewis acid sites [45]; and (5) dehydration of crotyl alcohol into butadiene occurs on the silanol groups [10,46]. 

The catalytic activity of catalysts for the direct ETB reaction was measured in a stainless-steel fixed-bed reactor with an internal diameter of 6 mm. Typically, a catalyst of 0.25 g was mixed with 1.25 g of quartz sand and loaded into the reactor, then pretreated at a nitrogen flow ratio of 20 mL·min^−1^ at 450 °C for 1 h. After cooling to reaction temperature (325–425 °C), the ethanol (95 vol%) was introduced into the reactor by a syringe pump with a flow ratio of 0.12–0.48 mL·h^−1^ and nitrogen as carrier gas (20 mL·min^−1^), corresponding to the weight hourly space velocity (WHSV) of ethanol of 0.38–1.53 h^−1^. The catalytic reaction was performed time-on-stream (TOS) for 6 h, and the products were analyzed via an online gas chromatograph (GC) Shimadzu GC-14B with a DB-1 capillary column (30 m × 0.25 mm × 0.25 μm) and a flame ionization detector (FID).

The calculations of ethanol conversion, product selectivity, butadiene yield, and space–time yield (STY) are defined as follows:

*X_EtOH_* (%) = (*n_EtOH,in_* − *n_EtOH,out_*)/*n_EtOH,in_* × 100 (1)

where *X_EtOH_* is the ethanol conversion; *n_EtOH_*_,*in*_ is the mole number of ethanol introduced into the reactor; and *n_EtOH_*_,*out*_ is the mole number of ethanol in the reaction products.

*S_i_* (%) = *A_i_f_i_*/∑*A_i_f_i_* × 100 (2)

where *S_i_* is the selectivity of product *i*; *A_i_* is the peak area of product *i*; and *f_i_* is the correction factor for quantitative product *i*.

*BD yield* (%) = *X_EtOH_* × *S_BD_* × 100 (3)

*STY* (g∙kg_cat_^−1^∙h^−1^) = (*X_EtOH_* × *n_EtOH_* × *S_BD_* × *M_BD_* × 10^3^)/(2 × *W_cat._*) (4)

where *STY* is the space–time yield; *n_EtOH_* is the mole number of ethanol introduced into the reactor per hour (mol∙h^−1^); *S_BD_* is the selectivity of product butadiene; *M_BD_* is the molar mass of butadiene (54.1 g∙mol^−1^); and *W_cat._* is the weight of the catalyst (g).

## 4. Conclusions

Here, a series of efficient multifunctional lignin-derived carbon-modulated ZnZr/SiO_2_ catalysts were prepared using the wet-impregnation method combined with a sample hydrothermal treatment of lignin, and the reaction performance of the direct ETB was evaluated. The 1%L-ZnZr/SiO_2_ catalyst exhibited an outstanding ethanol conversion of 98.4% and a butadiene selectivity of 55.5% at 400 °C and 0.77 h^−1^, and it also achieved excellent stability over 50 h of reaction. The characterization results revealed that lignin-derived carbon can efficiently modulate the acid–base properties of ZnZr/SiO_2_ catalysts to achieve great reaction performance in the direct ETB process. Moreover, the ethanol-TPSR results revealed that the rate-determining step was the aldol condensation of acetaldehyde in the direct ETB process. It also demonstrated that the synergy of multiple active sites could effectively boost butadiene synthesis from ethanol. This study provided a simple and universal catalyst design not only for the direct ETB process but also for other tandem reactions.

## Figures and Tables

**Table 1:** The acid properties of the ZnZr/SiO_2_ and m%L-ZnZr/SiO_2_ catalysts.

Catalysts	Acid Amount (mmol/g)							LAS Amount(μmol·g^−1^)
	T (°C)	Wa	T (°C)	Sa	Ta	Wa/Ta	Sa/Ta	
ZnZr/SiO_2_	160	0.15	347	0.10	0.25	0.60	0.39	85.0
0.2%L-ZnZr/SiO_2_	166	0.19	402	0.34	0.53	0.36	0.65	-
1%L-ZnZr/SiO_2_	172	0.20	406	0.34	0.54	0.37	0.64	65.7
3%L-ZnZr/SiO_2_	166	0.19	413	0.42	0.61	0.31	0.68	-
5%L-ZnZr/SiO_2_	162	0.18	415	0.59	0.76	0.24	0.77	-

Weak acid (Wa), strong acid (Sa), total acid (Ta).

**Table 2:** The base properties of the ZnZr/SiO_2_ and m%L-ZnZr/SiO_2_ catalysts.

Catalysts	Base Amount (mmol/g)						
	T (°C)	Wb	T (°C)	Sb	Tb	wb/Tb	Sb/Tb
ZnZr/SiO_2_	119	0.13	323	0.25	0.38	0.34	0.66
0.2%L-ZnZr/SiO_2_	121	0.08	345	0.56	0.64	0.13	0.87
1%L-ZnZr/SiO_2_	124	0.10	352	0.52	0.61	0.16	0.84
3%L-ZnZr/SiO_2_	126	0.08	365	0.74	0.82	0.10	0.90
5%L-ZnZr/SiO_2_	130	0.08	371	0.84	0.92	0.09	0.92

Weak basic (Wb), strong basic (Sb), total basic (Tb).

**Table 3:** Catalytic performance of ZnZr/SiO_2_ and m%L-ZnZr/SiO_2_ catalysts for the direct ETB process.

Catalysts	EtOH Conv. (%)	Product Sel. (%)						BD Yield(%)	STY (g·kg_cat_^−1^·h^−1^)
		BD	C2=	AcH	DEE	BuOH	Others		
ZnZr/SiO_2_	82.5	46.4	6.4	23.2	2.4	2.3	19.3	38.3	173
0.2%L-ZnZr/SiO_2_	79.6	49.9	10.1	19.9	2.3	1.9	15.9	39.7	180
1%L-ZnZr/SiO_2_	84.1	52.2	9.2	24.1	1.9	2.4	10.2	43.9	198
3%L-ZnZr/SiO_2_	80.0	50.4	8.9	24.9	2	2.7	11.1	40.3	182
5%L-ZnZr/SiO_2_	69.7	43.1	5.9	27.8	1.6	2.9	18.7	30.0	136

Reaction conditions: catalyst = 0.25 g, T = 375 °C, WHSV = 0.77 h^−1^, and TOS = 6 h. Ethanol, butadiene, ethylene, acetaldehyde, diethyl ether, 1-butanol, and space–time yield are abbreviated as EtOH, BD, C2=, AcH, DEE, BuOH, and STY, respectively.

## Data Availability

Not applicable.

## Supplementary Materials

The following supporting information can be downloaded at: https://www.mdpi.com/article/10.3390/molecules28186632/s1, Figure S1: SEM images of the catalysts (a) ZnZr/SiO_2_, (b) 0.2%L-ZnZr/SiO_2_, (c) 1%L-ZnZr/SiO_2_, (d) 3%L-ZnZr/SiO_2_, (e) 5%L-ZnZr/SiO_2_; Figure S2: Picture of the catalysts (a) ZnZr/SiO_2_, (b) 0.2%L-ZnZr/SiO_2_, (c) 1%L-ZnZr/SiO_2_, (d) 3%L-ZnZr/SiO_2_, (e) 5%L-ZnZr/SiO_2_; Table S1: Porous properties of ZnZr/SiO_2_ and m%L-ZnZr/SiO_2_ catalysts; Table S2: Catalytic performance of ZnZr/SiO_2_ and m%L-ZnZr/SiO_2_ catalysts for the direct ETB conversion; Table S3: Catalytic performance of ZnZr based catalysts in the ETB process; Table S4: Porous properties of various ZnZr based catalysts.
