# Supplementary material for: Tuning the Acid–Base Properties of Lignin-Derived Carbon Modulated ZnZr/SiO2 Catalysts for Selective and Efficient Production of Butadiene from Ethanol
Source: Molecules. 2023 Sep 15;28(18):6632. doi: 10.3390/molecules28186632 (PMC10536710; doi:10.3390/molecules28186632)
Supplement: Supplementary file 1 [file molecules-28-06632-s001.zip › molecules-2500624-supplementary.pdf]

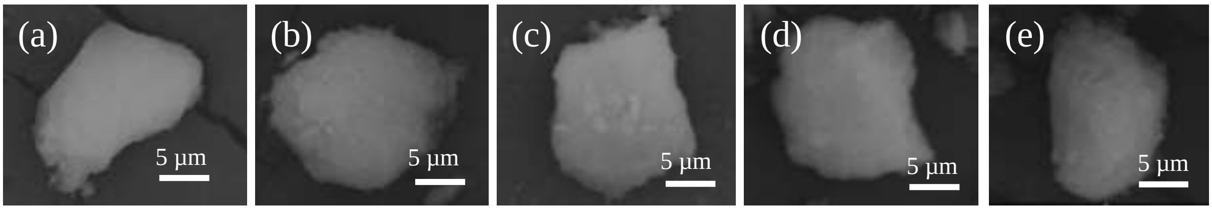

Figure S1. SEM images of the catalysts (a) $ZnZr/SiO_2$, (b) 0.2%L-$ZnZr/SiO_2$, (c) 1%L-$ZnZr/SiO_2$, (d) 3%L-$ZnZr/SiO_2$, (e) 5%L-$ZnZr/SiO_2$.

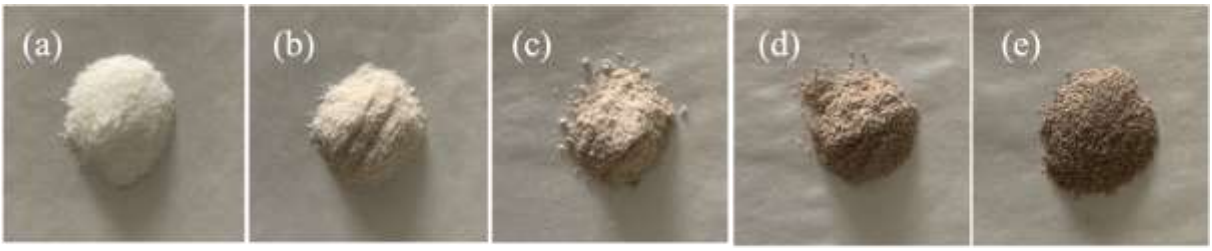

Figure S2. Picture of the catalysts (a) ZnZr/SiO_2, (b) 0.2%L-ZnZr/SiO_2, (c) 1%L-ZnZr/SiO_2, (d) 3%L-ZnZr/SiO_2, (e) 5%L-ZnZr/SiO_2.

Table S1. Porous properties of $ZnZr/SiO_2$ and m%L-$ZnZr/SiO_2$ catalysts.

Catalysts	S_{BET} [a] (m^2/g)	V_{total} [b] (cm^3/g)	V_{micro} [c] (cm^3/g)	V_{meso} [d] (cm^3/g)	D_{micro} [e] (nm)	D_{meso} [f] (nm)
$ZnZr/SiO_2$	171.9	1.16	0.06	1.10	1.06	27.04
0.2%L-$ZnZr/SiO_2$	176.3	0.93	0.07	0.86	1.04	23.07
1%L-$ZnZr/SiO_2$	170.4	0.89	0.06	0.83	1.06	21.84
3%L-$ZnZr/SiO_2$	160.4	0.85	0.06	0.79	1.04	22.67
5%L-$ZnZr/SiO_2$	151.2	0.80	0.06	0.74	1.03	22.60

[a] Calculated using the BET model. [b] Calculated at P/P_0=0.99. [c] Cumulative micropore volume with the pore size <2.0 nm using the HK model. [d] Cumulative mesoporous volume with the pore size (2.0 nm-50.0 nm) using the BJH model. [e] Average diameter of micropores were calculated using the HK model. [f] Average diameter of mesopores were calculated using the BJH model.

Table S2. Catalytic performance of ZnZr/SiO_2 and m%L-ZnZr/SiO_2 catalysts for the direct ETB conversion.

Temperature (°C)	EtOH conv. (%)	Product sel. (%)						BD yield (%)	STY ($g \cdot kg_{cat}^{-1} \cdot h^{-1}$)
		BD	$C_2^=$	AcH	DEE	BuOH	Others		
350	36.3	41.1	7.5	38.5	3.3	4.2	5.4	14.9	67
375	84.1	52.2	9.3	24	2.3	2.0	10.2	43.9	198
400	98.4	55.5	11.0	14.8	1.6	2.3	14.8	54.6	247
425	100	53.5	12.9	10.4	1.5	2.0	19.7	53.5	242

Reaction conditions: catalyst = 0.25 g, T = 375 °C, WHSV = 0.77 h^{-1}, and TOS = 6 h. The ethanol, butadiene, ethylene, acetaldehyde, diethyl ether, 1-butanol, and space-time-yield were abbreviated as EtOH, BD, $C_2^=$, AcH, DEE, BuOH, and STY, respectively.

Table S3. Catalytic performance of ZnZr based catalysts in the ETB process.

WHSV (h^{-1})	EtOH conv. (%)	Product sel. (%)						BD yield (%)	STY ($g \cdot kg_{cat}^{-1} \cdot h^{-1}$)
		BD	$C_2^=$	AcH	DEE	BuOH	Others		
0.38	99.4	52.7	11.6	16.6	0.5	1.9	16.7	52.3	117
0.77	98.4	55.5	10.7	15.7	1.1	2.3	14.8	54.6	247
1.15	84.1	52.2	9.3	24.0	2.3	2.0	10.2	43.9	296
1.53	79.8	46.6	19.9	15.6	4.8	3.0	10.1	37.2	168

Reaction conditions: catalyst = 0.25 g, T = 400 °C, WHSV = 0.77 h^{-1}, and TOS = 6 h. The ethanol, butadiene, ethylene, acetaldehyde, diethyl ether, 1-butanol, and space-time-yield were abbreviated as EtOH, BD, $C_2^=$, AcH, DEE, BuOH, and STY, respectively.

Table S4. Porous properties of various ZnZr based catalysts.

Catalysts	S_{BET} [a] (m^2/g)	V_{total} [b] (cm^3/g)	V_{micro} [c] (cm^3/g)	V_{meso} [d] (cm^3/g)	D_{micro} [e] (nm)	D_{meso} [f] (nm)
fresh 1%L-ZnZr/SiO_2	170.4	0.89	0.06	0.83	1.06	21.84
spent 1%L-ZnZr/SiO_2	132.7	0.84	0.06	0.78	1.06	26.90

[a] Calculated using the BET model. [b] Calculated at P/P_0=0.99. [c] Cumulative micropore volume with the pore size <2.0 nm using the HK model. [d] Cumulative mesoporous volume with the pore size (2.0 nm-50.0 nm) using the BJH model. [e] Average diameter of micropores were calculated using the HK model. [f] Average diameter of mesopores were calculated using the BJH model.
